# The Effect of High-Intensity Interval Training (HIIT) on Brain-Derived Neurotrophic Factor Levels (BNDF): A Systematic Review

**DOI:** 10.3390/brainsci15010034

**Published:** 2024-12-30

**Authors:** Milosz Mielniczek, Tore Kristian Aune

**Affiliations:** Sport and Human Movement Science Research Group (SaHMS), Department of Sport Science, Nord University, 7600 Levanger, Norway; milosz.msg@gmail.com

**Keywords:** neuroplasticity, cognition, endurance, intensity, adaptation

## Abstract

Background/Objectives: High-intensity interval training (HIIT) alternates short periods of intense exercise with recovery, effectively enhancing cardiorespiratory fitness, endurance, and strength in various populations. Concurrently, brain-derived neurotrophic factor (BDNF) supports neuronal resilience and activity-dependent plasticity, which are vital for learning and memory. This study aims to systematically review changes in BDNF levels in response to HIIT, with three primary objectives: evaluating the benefits of HIIT for BDNF modulation, assessing methodological quality and the risk of bias in reviewed studies, and identifying patterns in BDNF response based on HIIT protocols and population characteristics. Methods: Comprehensive database searches were conducted in PubMed and SPORTDiscus to identify relevant studies published up to April 2024. Given the diversity in study designs and outcomes, a narrative synthesis was performed rather than a meta-analysis. Bias was evaluated using visualization tools such as RobVis, and the review was conducted by a single researcher, which may limit its comprehensiveness. Results: Twelve studies met the inclusion criteria, with most indicating significant increases in BDNF levels post-HIIT, suggesting HIIT’s potential to enhance neuroplasticity and cognitive functions. However, variations in BDNF responses were observed across different HIIT protocols and study populations. Some studies reported decreases or no change in BDNF levels, reflecting the complex regulation of BDNF influenced by factors such as exercise intensity, duration, and individual variability. Conclusions: HIIT shows promise as an intervention for increasing BDNF levels, with potential benefits for brain health and cognitive function. These findings underscore the need for further research to confirm the optimal conditions under which HIIT can effectively enhance neurological outcomes. Future studies should explore standardized HIIT protocols and the long-term impact of HIIT on BDNF and neuroplasticity.

## 1. Introduction

Physical activity is well-known for its beneficial effects on both physical and mental health [1,2,3]. Moreover, specific types of exercise, such as high-intensity interval training (HIIT), have been shown to produce particularly positive outcomes for cognitive function [4,5,6]. Research also indicates that HIIT enhances cardiorespiratory fitness in both healthy individuals and those with physical health disorders [7,8,9,10].

The human brain has an incredible ability to adapt and change, known as neuroplasticity. This skill allows the brain to reconstruct its structures and functions by forming new neural connections in response to learning new things, gaining new experiences, and changes in the environment around it. Brain-derived neurotrophic factor (BDNF), discovered in 1982, is a protein that supports neuronal survival, differentiation, and synaptic plasticity. BDNF is an essential part of long-term potentiation, a mechanism that allows us to learn new things and memorize [11,12,13,14,15].

BDNF is considered to have therapeutic potential, making it a focus in research and in this case, regarding the effects of exercise on its modulation [16,17,18]. Some studies have shown that HIIT could optimize BDNF levels, adding to previously mentioned cognitive functions, such as learning and memory [17,18,19,20,21]. Based on the presented considerations and previous research, these findings could position HIIT as a promising form of exercise for cognitive health. Though its impact on BDNF needs to be looked into more closely.

HIIT is characterized by alternating short bursts of intense activity, typically performed at 80–90% of maximal heart rate, with periods of low-intensity recovery or rest. This training method allows us to direct focus on cardiovascular fitness, endurance, and metabolic rate in a relatively short amount of time [4,6,10]. However, despite its promising effects, the existing research on HIIT’s influence on BDNF remains limited, particularly regarding its long-term effects and responses. This underscores the need for more studies that focus on understanding the full potential physical activity has in enhancing brain health and cognitive function.

Understanding how different types of physical activities influence BDNF levels could even have an impact on general public health, especially in older populations which are at greater risk of cognitive decline [22]. As the global population continues to age, finding effective and affordable ways to support brain health is becoming increasingly important. Physical exercise presents a promising option, as it could not only improve one’s physical fitness but also offer protection against neurodegenerative diseases like Parkinson’s [23]. By potentially enhancing BDNF levels, HIIT could improve quality of life while reducing healthcare costs associated with brain-related diseases.

This study aims to systematically review changes in BDNF levels in response to HIIT, focusing on two primary objectives: synthesizing existing research to evaluate HIIT’s impact on BDNF modulation and identifying patterns in BDNF response based on population characteristics and HIIT protocols.

## 2. Materials and Methods

### 2.1. Eligibility Criteria

A strict inclusion and exclusion criteria was applied to ensure reliable and applicable findings. This review included studies with adult participants aged 18 years or older. To align with demographic conventions and acknowledge potential differences in physiological responses to training, participants were categorized as younger adults (18–64 years) and older adults (65 years and above) where applicable. Included studies focused exclusively on HIIT as the exercise intervention, with clearly defined session duration and frequency. Reporting changes in BDNF levels was mandatory to allow for the direct assessment of HIIT’s effects. Only peer-reviewed studies written in English and with full-text availability were considered to meet the scholarly standards and ensure accessibility. Studies were excluded if they did not use HIIT as the primary intervention or failed to report BDNF levels. Meta-analyses and systematic reviews were also excluded to maintain originality in the synthesis of research.

### 2.2. Information Sources

A search was conducted in the PubMed and SPORTDiscus electronic databases. These databases were chosen because they provided extensive coverage of studies relevant to exercise physiology, neurobiology, and related fields, making them well-suited for this systematic review. While additional databases could have been included, the focus on these two ensured a targeted and manageable scope for this review. Future studies may benefit from including broader database searches to capture additional literature. The reference lists of the included studies were hand-searched to ensure the completeness of the search. The search was conducted up to the end of April 2024, with no restrictions on the start date to capture as much relevant literature as possible. While missing data were identified in some studies (e.g., training duration in three studies listed in Table 1), the authors of these studies were not contacted to retrieve the missing information. Future systematic reviews should consider contacting study authors as a strategy to address such gaps.

### 2.3. Search Strategy

The search strategy for this systematic review was designed to identify studies that explore the effects of HIIT on BDNF. To ensure comprehensive coverage of the literature, a combination of specific search terms and words was used. These terms were “High-Intensity Interval Training”, “HIIT”, “Brain-Derived Neurotrophic Factor”, and “BDNF”. Boolean operators such as AND and OR were used to connect these terms. An illustrative example of how these terms were applied in a search string for PubMed is as follows: (“High-Intensity Interval Training” OR “HIIT”) AND (“Brain-Derived Neurotrophic Factor” OR “BDNF”).

### 2.4. Selection Process

**Figure 1:**
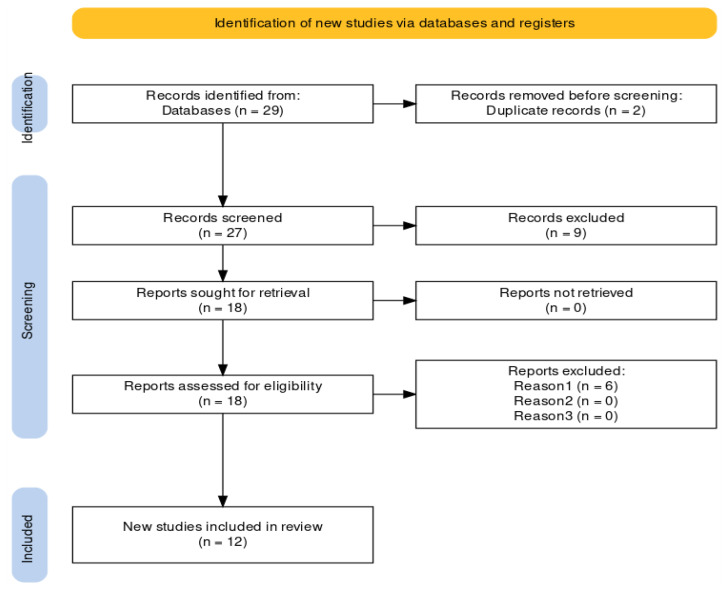
PRISMA flow diagram of search results/of study selection.

The selection process for this systematic review was executed in two stages. In the first phase, title screening and abstract screening were conducted independently by two authors (M.M. and T.K.A.) to determine if they met the inclusion criteria; discrepancies during this phase were resolved through discussion. This step filtered out studies that did not match the research needs. In the second phase, full-text articles were reviewed by both authors, with a third independent reviewer available for resolving conflicts, though no major disagreements arose. The complete selection process, from the initial number of studies screened to the final number included in the review, is reported and shown in Figure 1. PRISMA flow diagram of search results on the Section 3. This PRISMA flow diagram provides a summary of the information flow through the different stages of the systematic review, including screening, eligibility checks, and study inclusion.

### 2.5. Data Collection Process

Data extraction was performed by one author (M.M.) using a standardized data extraction form. The extracted data were cross-checked for accuracy and consistency by the second author (T.K.A.). This process ensured the reliability of the collected data and minimized the risk of errors. The form includes participant data such as age, sex, and the size of the sample; specifics of the study design included the type of HIIT intervention conducted, together with their duration and frequency, and a summary of the findings focusing on changes in BDNF levels. All included studies assessed BDNF levels using the enzyme-linked immunosorbent assay (ELISA) method. ELISA is a widely used technique that utilizes antibody–antigen interactions and enzyme-mediated signal detection to quantify protein levels in biological samples with high specificity and sensitivity. For more details on the principles and applications of the ELISA method, see [33].

### 2.6. Study Risk of Bias Assessment

In the present systematic review, the risk of bias for each included study was assessed using RobVis, a web-based application designed specifically for visualizing risk-of-bias assessments within systematic reviews. This tool was chosen for its ability to communicate the results of bias assessments through its visualization features.

**Figure 2:**
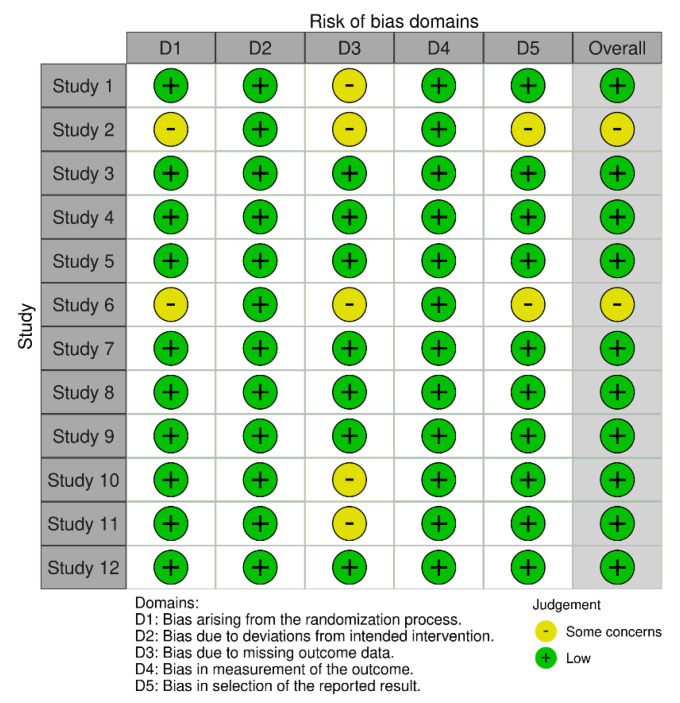
Risk of bias assessment for the included studies.

**Figure 3:**
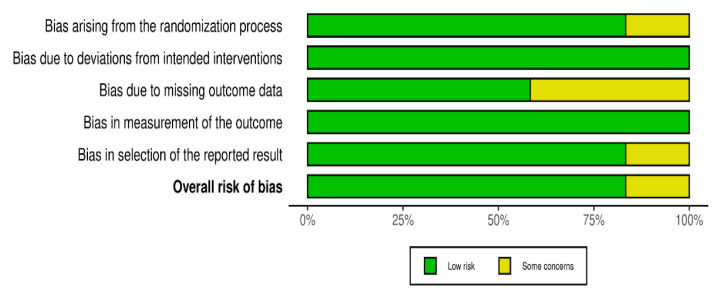
Graph bar results of the risk of bias assessment.

This tool provides two main types of visual outputs. The first one, “Traffic Light” plot (Figure 2), displays the domain-level judgments for each study, using a color-coded system where green indicates a low risk of bias, yellow indicates some concerns, and red indicates a high risk of bias. This visualization allows for an immediate grasp of the areas where biases may exist within and across studies. The second type of output is the weighted bar plots (Figure 3). These plots show the distribution of risk-of-bias judgments within each bias domain across all included studies, weighted by the importance of each domain. This helps in understanding the overall landscape of biases that might affect this review’s findings.

### 2.7. Synthesis Methods

The synthesis of findings was conducted using a narrative synthesis method. Given the variability in study designs, populations, and interventions among the included studies, a quantitative synthesis (meta-analysis) was not feasible. Instead, a narrative approach was employed to describe and interpret the findings from each study.

Studies were grouped and compared based on key characteristics such as participant demographics, specific HIIT protocols, and outcome measures. The narrative synthesis provided a structured summary of the evidence, highlighting patterns and variations.

The extracted data, including study design, population characteristics, intervention protocols, and outcomes, were tabulated and synthesized by M.M. The synthesis was reviewed and validated by T.K.A. to ensure the accurate interpretation and reporting of the findings.

## 3. Results

### 3.1. Study Selection

The study selection process is summarized in Figure 1, where a PRISMA flow diagram provides a transparent and detailed numerical breakdown of the screening and selection process. A total of 29 records were identified through the database searches, with 27 records proceeding to title and abstract screening after duplicate removal. Following full-text assessment, six studies were excluded because they were not peer-reviewed, resulting in twelve studies being included in the final synthesis.

### 3.2. Risk of Bias in Studies

Figure 2 illustrates the risk of bias across five domains (D1–D5) for each study included in this systematic review. Judgements are categorized as ’Low’ risk (green) and ’Some concerns’ (yellow). 

The risk of bias assessment across the 12 studies included reveals a generally favorable methodological quality, though with some areas of concern. Most studies described a clear randomization process, ensuring the systematic and unbiased allocation of participants to intervention groups, with the exception of two studies that did not provide detailed descriptions of the randomization method, leading to some uncertainty regarding the allocation process. All studies reported standardized and monitored interventions, indicating that the HIIT protocols were consistently applied and adhered to as planned. While the majority of the studies adequately addressed the issue of missing data, either by reporting minimal dropouts or by having protocols in place to handle missing data appropriately, three studies did not provide sufficient details on how missing data were managed, raising potential concerns about the impact of incomplete data on the study’s results.

All studies utilized standardized and validated methods for measuring BDNF levels and other outcomes, ensuring consistency and reliability in the reported results. Most studies appeared to report on all expected outcomes based on their documented methodologies, although two studies lacked explicit information on whether all measured outcomes were reported, leaving room for potential selective reporting. Overall, eight studies were assessed to have a low risk of bias, indicating robust methodological quality and reliable findings, while four studies presented some concerns in certain domains, primarily related to randomization processes and the handling of missing data, which could affect the overall reliability of their findings, as shown in Figure 3.

### 3.3. Study Characteristics

The results of each study examining the effects of HIIT on BDNF levels are consolidated in Table 1. This table serves as a central component of this review, providing a clear and organized presentation of the key findings from each included study. It is noteworthy to mention that all of the studies assessed BDNF using the ELISA method. Participants were grouped into younger adults (18–64 years) and older adults (≥65 years) to reflect potential differences in neuroplasticity and physiological responses to HIIT. These distinctions are essential for interpreting age-related variations in BDNF responses.

The systematic review analyzed 12 studies with diverse sample sizes, gender compositions, age ranges, test designs, durations, frequencies, and intervention types. Regarding gender, five studies focused exclusively on male participants [25,28,31], one on female participants [24], and six included mixed-gender samples [4,26,27,29,30,32]. Participant ages ranged from young adults (mean age 18.5 ± 4.6 years in [26]) to older adults (mean age 64.8 ± 3.9 years in [29]). Study designs varied, with eight employing randomized controlled trials [24,25,26,27,29,30,31,32], three using experimental designs [20,25,28], and one employing a cross-sectional approach [28].

The duration of the studies ranged from four weeks [24] to six months [26]. Training session frequency varied, with most studies conducting sessions two to three times per week [24,25,26,27,29]. Other frequencies included four experimental sessions spaced one week apart [20], four sessions with intervals longer than one week [21], and multiple sessions conducted over different days [4].

Regarding the types of interventions, the studies predominantly utilized high-intensity interval training (HIIT) with varying protocols; cycling HIIT was used in studies by [24,25,26]; running or walking HIIT was employed in studies by [4,20,27]; and mixed or other forms of HIIT, including treadmill and seated stepper, were applied in studies by [21,30], ergometer in [31], and alternating intensities in [32]. Additionally, ref. [25] combined HIIT with strength training. This variety in HIIT protocols, participant demographics, and training regimens provides a comprehensive overview of the current research landscape on HIIT and BDNF levels.

### 3.4. Results of Individual Studies

The results examining the effects of HIIT on BDNF levels, also depicted in Table 1, which varied across studies. Four studies [21,24,28,32] reported significant increases in BDNF levels following HIIT interventions, highlighting the potential of HIIT to enhance neuroplasticity. Two studies [25,32] observed a decrease in BDNF levels post-training, suggesting a possible adaptation or regulation mechanism over time. Three studies [20,26,27] found that BDNF levels increased immediately after HIIT but returned to baseline shortly thereafter, indicating a short-term effect. In contrast, two studies found no significant change in BDNF levels, implying that factors such as intensity, duration, or individual differences might influence the outcomes [30,31]. Additionally, two other studies reported significant increases in BDNF, with the latter emphasizing long-term HIIT benefits in older adults [4,29]. Finally, one study noted varied effects depending on exercise intensity, further suggesting that BDNF response may be intensity-sensitive [20]. Overall, the evidence suggests that while HIIT can effectively increase BDNF levels, the magnitude and duration of these effects can vary based on training protocols, participant characteristics, and other contextual factors.

## 4. Discussion

The results of the present systematic review examining the effects of HIIT on BDNF levels varied significantly across the included studies, indicating a complex relationship influenced by multiple factors. Out of the twelve studies, four [21,24,28,32] reported significant increases in BDNF levels following HIIT intervention, highlighting the possibility of HIIT influencing neuroplasticity. This consistent finding across different cohorts suggests that HIIT can effectively stimulate BDNF production through increased heart rate and energy expenditure during intense exercise bursts followed by periods of rest.

Two of the studies [21,24] found significant BDNF increases in short training durations, emphasizing that the intensity of exercise plays an important role in triggering these responses. Reference [28] demonstrated that HIIT boosts BDNF even in elite athletes, while other study showed its benefits in stroke patients, suggesting broad applications in health and rehabilitation [32]. In contrast, two studies [25,32] observed a decrease in BDNF levels post-training, suggesting that factors such as exercise modality, duration, or individual variability may influence the neurotrophic response. Initially, HIIT may boost BDNF, but sustained high-intensity exercise could lead to feedback inhibition, where the body reduces BDNF production to maintain balance and prevent potentially negative effects of high levels of BDNF. One study examined eight weeks of combining HIIT with strength training [25], and another one [32] conducted a three-month study on stroke patients; both found decreases in BDNF, likely due to prolonged physiological stress, which may override the neurotrophic benefits of exercise under certain conditions.

Three of the studies [20,26,27] found that BDNF levels increased immediately after HIIT but returned to baseline shortly thereafter. This suggests that the effects of HIIT on BDNF are short-term and may require continuous or repeated stimuli to maintain elevated levels. The immediate post-exercise increase is likely due to acute physiological stress, with the return to baseline indicating rapid regulatory mechanisms. Interestingly, two studies [30,31] found no significant change in BDNF levels, pointing to factors such as exercise intensity, duration, or individual differences. Variations in participants’ fitness levels, genetic predispositions, or specific protocols used might account for these differences in response, indicating that not all HIIT protocols or participant groups respond uniformly in terms of BDNF production. Two more studies [4,29] reported significant increases in BDNF, with one of them [29] particularly emphasizing the long-term benefits of HIIT in older adults. This suggests that older populations may experience more pronounced neurotrophic benefits from HIIT, potentially because the exercise acts as a greater relative stressor on their physiological systems compared to younger individuals. Moreover, one last study [20] noted varied effects depending on exercise intensity, suggesting that the BDNF response may be highly sensitive to the intensity of the exercise. Higher intensities might produce more significant increases in BDNF, but there may be an optimal intensity range where production is maximized without triggering negative feedback mechanisms.

Overall, the evidence indicates that while HIIT can effectively increase BDNF levels, the magnitude and duration of these effects are influenced by several factors, including training protocols, participant characteristics, and other contextual elements. These varying outcomes highlight the complexity of optimizing HIIT for enhancing neuroplasticity through BDNF modulation. Understanding the interplay between exercise intensity, duration, and individual differences is crucial for tailoring HIIT protocols to maximize their neurobiological benefits.

While this systematic review provides insights into the effects of HIIT on BDNF levels, several limitations within the included evidence must be acknowledged. The studies reviewed included a variety of designs, such as randomized controlled trials and observational studies. This diversity enhances the depth of data but also introduces variability in methodological quality, potentially affecting the comparability and broader applicability of the results. Additionally, most studies focused on the short-term effects of HIIT on BDNF levels, limiting insights into the sustained impacts on neuroplasticity and cognitive function. Despite the risk assessments, some studies presented potential biases related to participant selection and outcome reporting, which could skew the results and interpretations. Furthermore, the variability in HIIT intensity, frequency, and duration across studies poses challenges in recommending specific exercise regimens. This variability makes it difficult to draw definitive conclusions about the optimal HIIT protocol for increased BDNF production. These limitations require careful interpretation of the findings and underscore the need for future research. To accurately evaluate the long-term effects of HIIT on BDNF levels and neuroplasticity, it is crucial to conduct longitudinal studies by employing standardized measurement techniques and consistent HIIT protocols.

This systematic review was conducted with rigorous standards. However, it is crucial to acknowledge several limitations in this review process. The scope of the literature search may not have encompassed all relevant studies, particularly those published in languages other than English or in less accessible journals, potentially limiting the comprehensiveness of the analyzed data. The selection of studies and the extraction of data involved subjective decisions that could introduce selection bias or errors in data interpretation. Additionally, despite efforts to assess the quality and risk of bias within the included studies, such assessments remain subjective and could influence the conclusions drawn from this review. Furthermore, this review was conducted by two individuals, which may have restricted the scope and thoroughness of the search and analysis. To address these limitations, future updates could broaden the search criteria to include more databases and non-English publications, employ a larger team of reviewers or automated tools to minimize human error and bias, and consider incorporating quantitative synthesis methods as more standardized data become available.

Future primary studies should aim to address the gaps identified in this review by adopting standardized HIIT protocols to reduce variability in intensity, frequency, and duration. Investigating the long-term effects of HIIT on BDNF levels and neuroplasticity is critical, particularly through longitudinal studies across diverse populations. Specific subgroup analyses, such as age-specific and fitness-level-specific responses, could provide deeper insights into ways of personalizing HIIT interventions to better meet individual needs. 

## 5. Conclusions

This systematic review shows that high-intensity interval training (HIIT) has the potential to influence brain-derived neurotrophic factor (BDNF) levels, potentially linking it to enhanced neuroplasticity. However, the effects of HIIT on BDNF are influenced by factors such as exercise intensity, duration, and individual differences among participants. While some studies demonstrated substantial BDNF increases, others observed decreases or no change, indicating the complexity of BDNF regulation in response to HIIT. The findings suggest that optimal HIIT protocols need to be tailored to individual needs, considering the balance between intensity and duration to maximize neurobiological benefits. Further research is necessary to standardize HIIT protocols and fully understand the mechanisms underlying these varying responses.

## Figures and Tables

**Table 1:** Summary of studies’ characteristics.

First-Named Author (Year)	Sample Size	Sex	Age	Test Design	Duration	Frequency	HIIT Intervention	Main Results
Rentería (2020) [24]	17	Female	22 ± 1 years	RCT	4 weeks	3 times/week	Each HIIT session consisted of three to five cycling bouts of 30 s each at 80% MAP, followed by four minutes of recovery at 40% MAP.	HIIT significantly increased serum BDNF concentrations by approximately 12% compared to the control group.
Figueiredo (2019) [25]	21	Male	18 to 35 years	ED	8 weeks	3 times/week	Running on a treadmill for one minute at 100% speed VO_2_max, followed by one minute of passive recovery, repeated until completing 5 km.	HIIT combined with strength training led to a decrease in BDNF concentrations post-training compared to pre-training values.
Hebisz (2019) [26]	26	Mixed	18.5 ± 4.6 years	RCT	6 months	2 times/week	Three to four sets of maximal cycling, each set comprising four 30 s all-out repetitions interspersed with 90 s of recovery.	Significant decreases in BDNF relative to baseline values after 10 min after the first set and 60 min after the third set in the experimental group at the 2- and 6-month assessments. No significant changes in baseline BDNF were observed in either group.
Ji (2024) [20]	9	Male	24.0 ± 0.4 years	ED	4 weeks	1 time/week	Four 30 s bouts of “all-out” cycling (Wingate test) followed by 4 min of recovery.	Post-exercise BDNF was significantly higher after the HIIT session compared to other forms of exercise tested.
Kovacevic (2020) [27]	64	Mixed	60–88 years	RCT	12 weeks	3 times/week	Intervals of 4 min at 90–95% PHR, separated by 3 min recovery periods at 50–70% PHR.	No significant differences in BDNF levels were found between the groups.
Reycraft (2020) [21]	8	Male	23.1 ± 3 years	RCT	n.a.	4 times	Four 30 s “all-out” running bouts interspersed with 4 min rest periods.	Significant increases post-exercise compared to other forms of exercise. BDNF levels returned to baseline at 30 min and 90 min post-exercise.
Marquez (2015) [4]	29	Male	28 ± 5 years	RCD	n.a.	7 times	Alternating between 20 min and 1 min intervals at 90% MaxWR and 1 min rest periods.	BDNF levels increased during exercise protocols, peaking towards the end of exercise and returning to baseline levels 20 min post-exercise.
Buzdagli (2024) [28]	28	Male	24.43 ± 4.72 years	CS	n.a.	4 times	A total of 3 min at 85% VO_2_max, 2 min at 95% VO_2_max, and 1 min at 100% VO_2_max, with 1 min passive recovery between intensities. Repeated twice.	Immediately after HIIT, BDNF levels were significantly higher compared to control group and the group that performed another form of exercise. One hour after HIIT, BDNF levels returned to baseline levels.
Li (2021) [29]	29	Mixed	64.8 ± 3.9 years	RCT	12 weeks	3 times/week	A total of 4 × 3 min at 90% VO_2_max interspersed with 3 min at 60% VO_2_max.	BDNF increased significantly in the HIIT group.
Boyne (2019) [30]	16	Mixed	57.4 ± 9.7 years	WCD	3 weeks	3 times	Treadmill HIIT 30 s bursts at maximum tolerated speed, alternated with recovery periods. Seated stepper HIIT with similar bursts and recovery durations as HIT-Treadmill but performed on a seated stepper.	HIT-Treadmill showed a significant increase in BDNF. HIT-stepper showed an initial BDNF response similar to HIT-Treadmill.
Azuma (2015) [31]	12	Male	35 ± 6 years	RCT	16 weeks	2 times/week	Ergometer at >90% VO_2_ peak for 60 s, followed by 60 s of active rest. Each session included 8–12 sets.	No significant change in BDNF was observed after 16 weeks of HIIT. However, there was a positive association between baseline serum BDNF concentration and the percentage increase in peak watt after the intervention.
Hsu (2021) [32]	23	n.a.	55	RCT	3 months	2–3 times/week	Alternating 3 min at 80% VO_2_ peak and 3 min at 40% VO_2_ peak.	Significant increase in serum BDNF levels.

Notes. Abbreviations: RCT, randomized controlled trial; ED, experimental design; RCD, randomized crossover design; CS, cross-sectional study; WCD, within-participant crossover design; BDNF, brain-derived neurotrophic factor; HIIT, high-intensity interval training; n.a., not assessed; PHR, peak heart Rate. MAP, maximal aerobic power; MaxWR, maximum work rate; VO_2_max, maximal oxygen consumption.
